# Total hip arthroplasty for post-firearm hip arthritis complicated by coloarticular fistula: A case report

**DOI:** 10.1016/j.cjtee.2023.04.004

**Published:** 2023-04-15

**Authors:** Ahmed M. Abdelaal, Mohammad Kamal Abdelnasser, Mohamed MA. Moustafa, Ahmed Mohamed Ali, Haisam Atta, Ahmed A. Khalifa

**Affiliations:** a Orthopaedic and Traumatology Department, Assiut University Hospital, Assiut, Egypt; b General Surgery Department, Assiut University Hospital, Assiut, Egypt; c Radiology Department, Assiut University Hospital, Assiut, Egypt; d Orthopedic Department, Qena Faculty of Medicine and University Hospital, South Valley University, Qena, Egypt

**Keywords:** Hip firearm injury, Total hip arthroplasty, Secondary hip osteoarthritis, Coloarticular fistula

## Abstract

Hip firearm injuries are rare injuries that could lead to serious complications, such as posttraumatic hip arthritis and coloarticular fistula. We report a case of a 25-year-old male who sustained a pelvic injury caused by a single bullet which led to a bilateral acetabular fracture, concomitant with a colon injury treated on an emergency basis by a diverting colostomy; acetabular fractures were treated conservatively by traction. After the patient recovered from the abdominal injury, he was presented with bilateral hip pain and limited motion; plain radiographs showed bilateral hip arthritis with proximal migration of the femoral head and bilateral acetabular defect classified as Paprosky type ⅢA. Reconstruction of the hips was performed using the same technique: impaction bone grafting for acetabular defect reconstruction and a reversed hybrid total hip arthroplasty (THA) 6 months apart. The patient presented with loosening of the left THA acetabular cup 3 years later, which was revised; then he presented with a discharging sinus from the left THA with suspicion of coloarticular fistula, which was confirmed using CT with contrast material. A temporary colostomy and fistula excision were performed, and a cement spacer was applied to the hip. After clearing the infection, a final revision THA for the left hip was performed. Treating post-firearm hip arthritis by THA is challenging, especially in the situation of neglected cases with the presence of an acetabular defect. Concomitant intestinal injury increases the risk of infection with the possibility of coloarticular fistula formation, which could present later. Working with a multidisciplinary team is paramount.

## Introduction

Firearm injuries affecting the hip are reported to occur at a range of 2% – 7% of all extremity firearm injuries^1^; however, there is a scarcity of publications reporting the management options of such injuries.^1^, ^2^, ^3^, ^4^

Recent hip injuries or fractures caused by firearm injuries could be treated by open reduction and internal fixation if the patient's general condition allows^5^; however, if the gunshot caused an abdominal or intrapelvic injury (bladder, intestine, or vascular structures), the priority is to save the life of the patient^6^. For late presented injuries or if it was complicated by posttraumatic osteoarthritis (PTOA), hip arthrodesis or total hip arthroplasty (THA) are the options for management.^5^^,^^6^

Although hip joint coloarticular fistula is considered rare, it was reported could occur in patients with inflammatory bowel disease or bowel carcinomas; it also could occur as complications during revision THA, especially when the acetabular floor is perforated with protruding components^7^, ^8^, ^9^, ^10^, ^11^, ^12^, ^13^; however, coloarticular fistula was rarely reported as a complication of firearm hip injuries^14^.

We present a male case who developed bilateral hip PTOA secondary to firearm injury by a single bullet, treated by a sequential bilateral THA, which was complicated lately by a coloarticular fistula.

## Case report

**Fig. 1:**
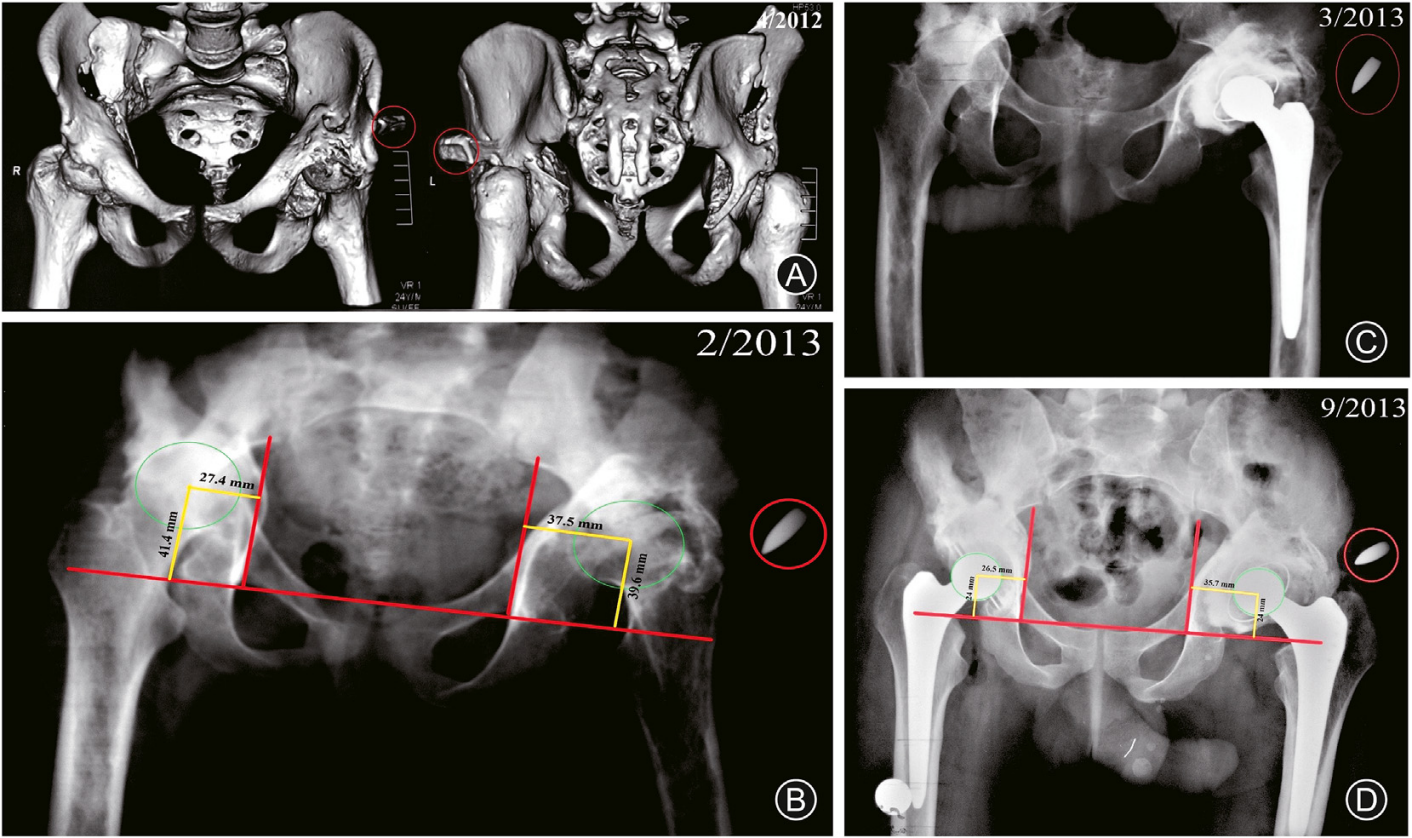
(A) Pelvis CT at the time of the patient's first presentation at the trauma unit showing bilateral iliac wing and acetabular fractures and a retained bullet (red circle). (B) Preoperative plain radiographs showing bilateral hip arthritis, proximal migration of the femoral head bilaterally and type ⅢA acetabular bone defect according to Paprosky classification. (C)& (D) Postoperative radiographs showing reconstruction of the hips bilaterally by impaction bone grafting and reversed hybrid total hip arthroplasty.

A 25-year-old male patient presented to the trauma unit of our hospital (a tertiary level 1 trauma center) in April 2012 with a history of recent (few hours) firearm injuries to the pelvic region. Following the advanced trauma life support protocol for initial management and assessment, the clinical evaluation revealed an inlet wound on the right hip region about 5 cm above the greater trochanter with no exit wound. The patient had a rigid abdomen, painful limited hip motion bilaterally, and the neurovascular examination of both lower limbs was free. Imaging studies in the form of urgent abdominal ultrasound and CT scan of the abdomen and pelvis revealed internal pelvic collection (suspected intestinal perforation) and bilateral iliac wing fractures with an extension of the fracture lines to the acetabulum (Fig. 1A). The patient was admitted for an emergency abdominal exploration which revealed an injury to the descending colon, and a diverting colostomy was performed. Debridement of the inlet wound was performed. Bilateral upper tibial skeletal traction was applied. The patient was admitted to the intensive care unit for a month, and re-anastomosis of the colon was performed 2 months later.

The patient was presented to the outpatient orthopedic clinic 10 months after the initial injury (in February 2013), complaining of progressive bilateral hip pain and limitation of motion (Harris hip score (HHS) was 36.9 for the right hip and 37.4 for the left hip); pelvis X-ray revealed bilateral arthritic hip with bilateral femoral heads proximal migration and bilateral acetabular defects which were classified according to the Paprosky classification system as type ⅢA on both sides (Fig. 1B).

A decision was made to perform a sequential bilateral THA 6 months apart; fresh frozen femoral head allografts were prepared for acetabulum reconstruction. Both surgeries were performed by the same surgeon under spinal anesthesia in a lateral decubitus position utilizing a modified direct lateral approach. A hybrid THA (cemented cup and cementless stem) bilaterally (the left hip was operated on in March 2013 and the right hip in September 2013), reconstruction of the acetabular defect was performed with impaction bone grafting (IBG) (Fig. 1C&D), the technique of which is well described in the literature.^15^

**Fig. 2:**
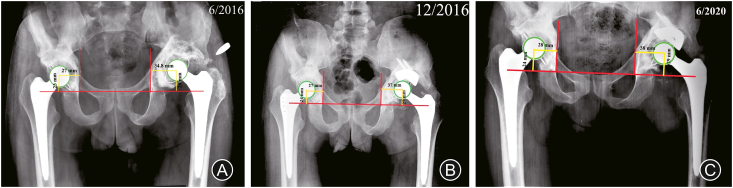
Follow-up radiographs. (A) Loosening and proximal migration of the left acetabular component; (B) Revision of the acetabular component by a cemented cup and a metal augment; (C) Septic loosening of the left acetabular cup.

**Fig. 3:**
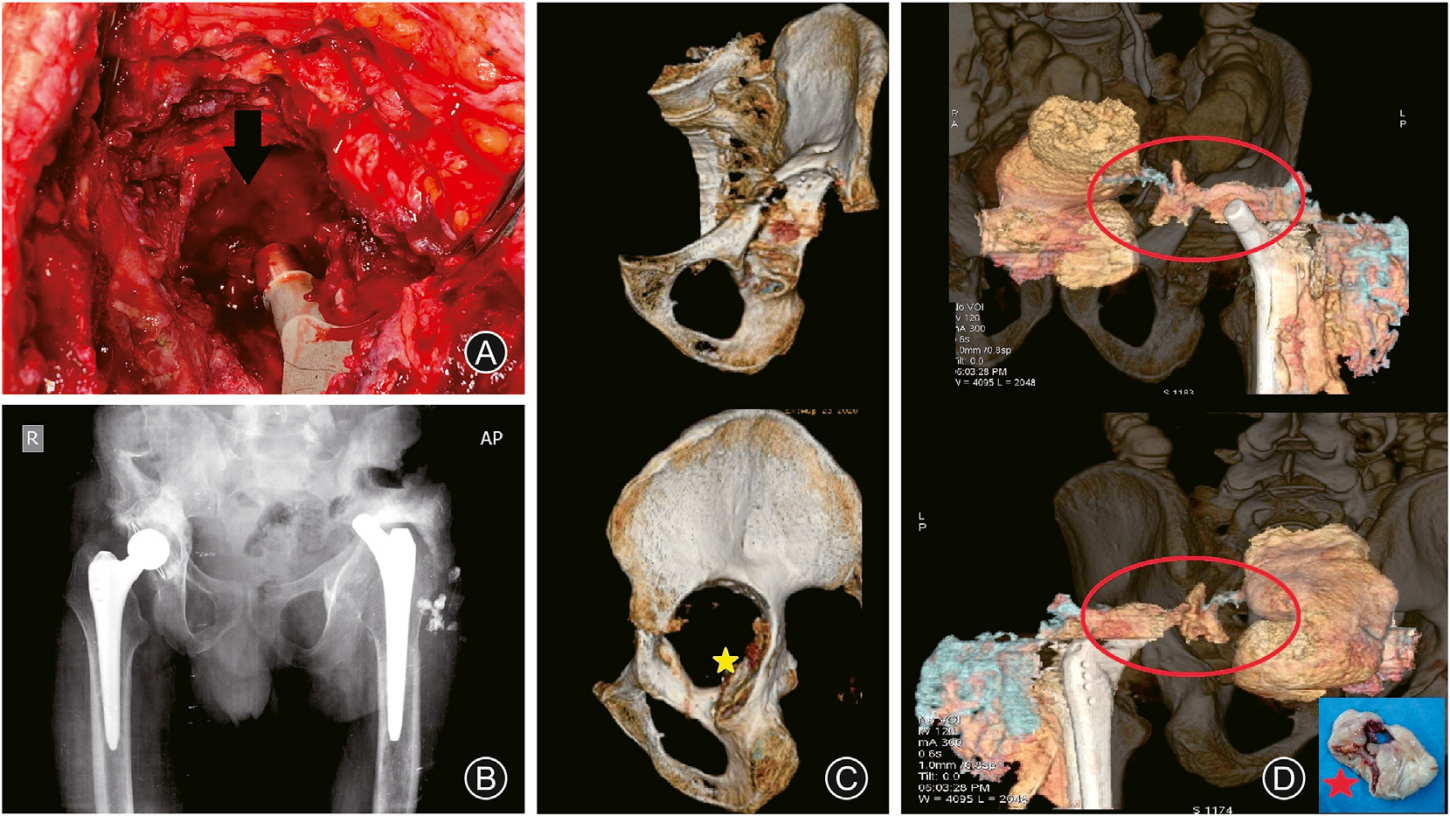
Detecting the presence of coloarticular fistula. (A) and (B) Intraoperative and postoperative images (radiographs and CT) showing acetabular floor deficiency type ⅡC (yellow asterix) and retained stem; (D) CT scan with contrast showing the coloarticular fistula (red circle) and after fistulas tract excision (red asterix).

In June 2016, the patient presented with progressive left THA pain; the plain radiographs showed loosening and proximal migration of the acetabular component. The patient was advised of revision surgery for the acetabular component. Revision of the acetabular cup and reconstruction of the acetabular defect using a tantalum metal augment was performed in December 2016; tissue cultures obtained during revision surgery, were negative for bacterial growth; the following few years were unremarkable. The patient presented in June 2020 complaining of recurrent attacks of left hip pain and a discharging sinus at the surgical wound site; radiographs showed loosening of the cup and proximal migration. At this time, the patient was diagnosed with septic loosening of the left THA (mainly the acetabular component) (Fig. 2). The decision was made to perform 2 stages of THA revision. During the first stage of surgery in September 2020 (entailing implants removal, debridement, and applying a cement spacer), the surgeon noticed an in and out movement of the acetabular floor with every patient's breath, as well as smelling fecal odor from the surgical field, so the surgeon only removed the cup, and the stem was left in place (Fig. 3A&B); cultures were obtained from the field (which later showed no growth) after consulting a general (colorectal) surgeon who advised an abdomen and pelvis CT with contrast, which revealed a colo-acetabular fistula, and the CT pelvis bone window showed central acetabular defect classified as type ⅡC according to Paprosky classification (Fig. 3C&D).

**Fig. 4:**
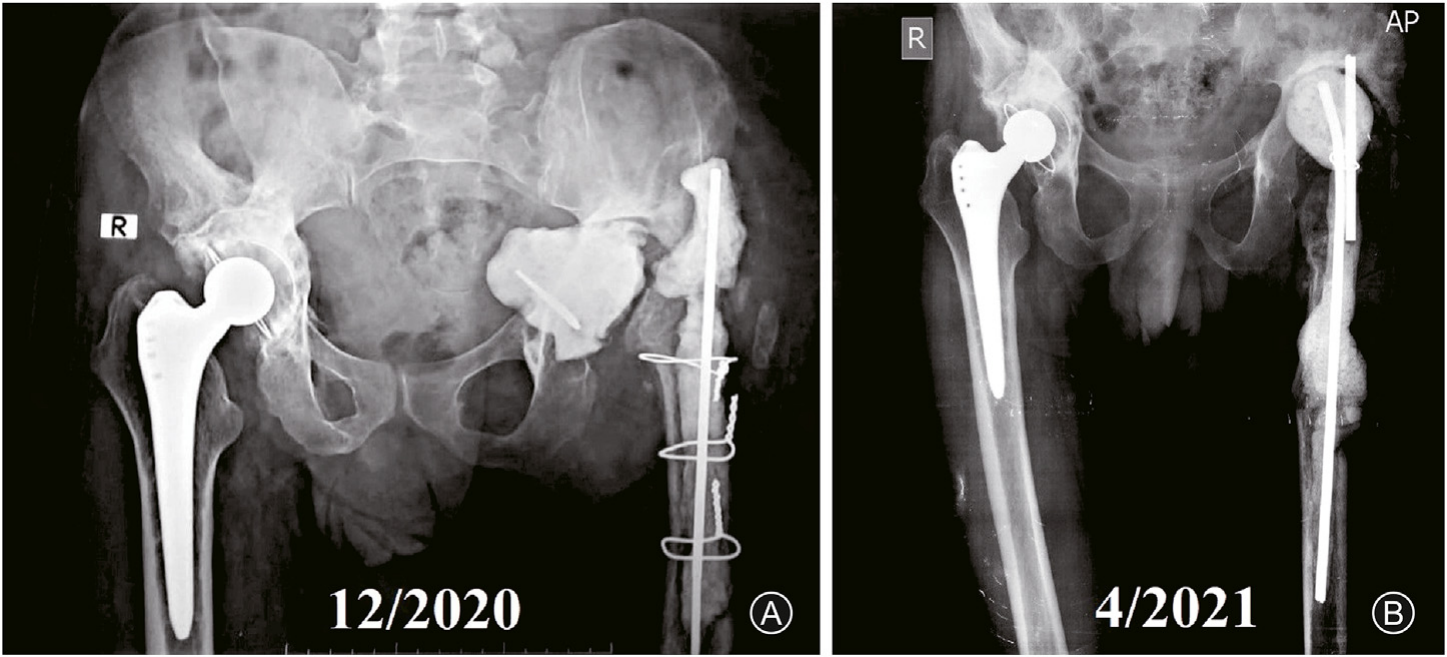
Debridement and cement spacer application. (A) Non-articulating spacer; (B) Articulating spacer.

**Fig. 5:**
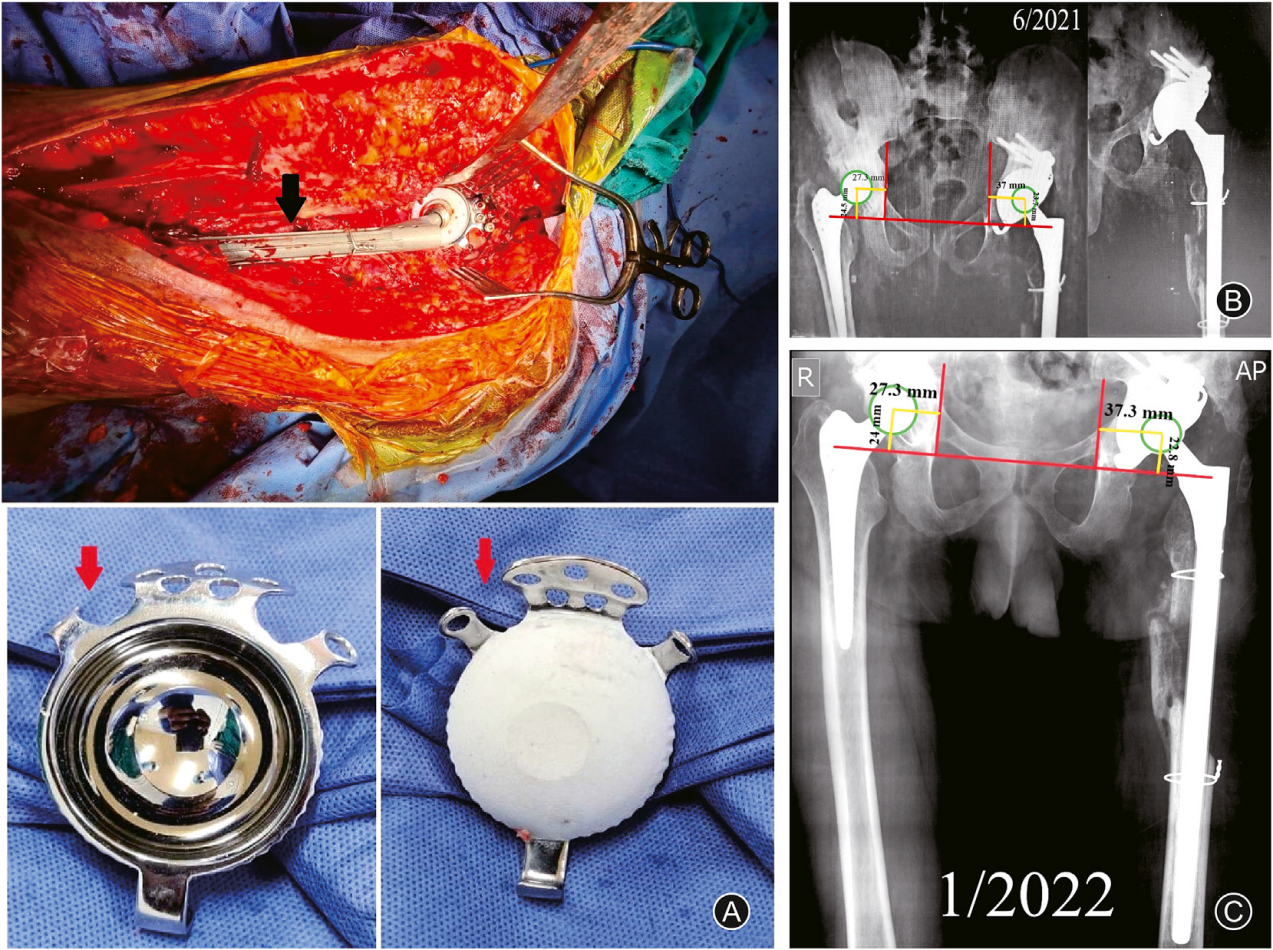
Last revision of the left total hip arthroplasty. (A) Intraoperative images showing the Wagner cementless stem (black arrowhead) and cementless dual mobility cup (red arrowhead); (B) Immediate postoperative radiographs; (C) Eight months follow-up radiographs.

**Fig. 6:**
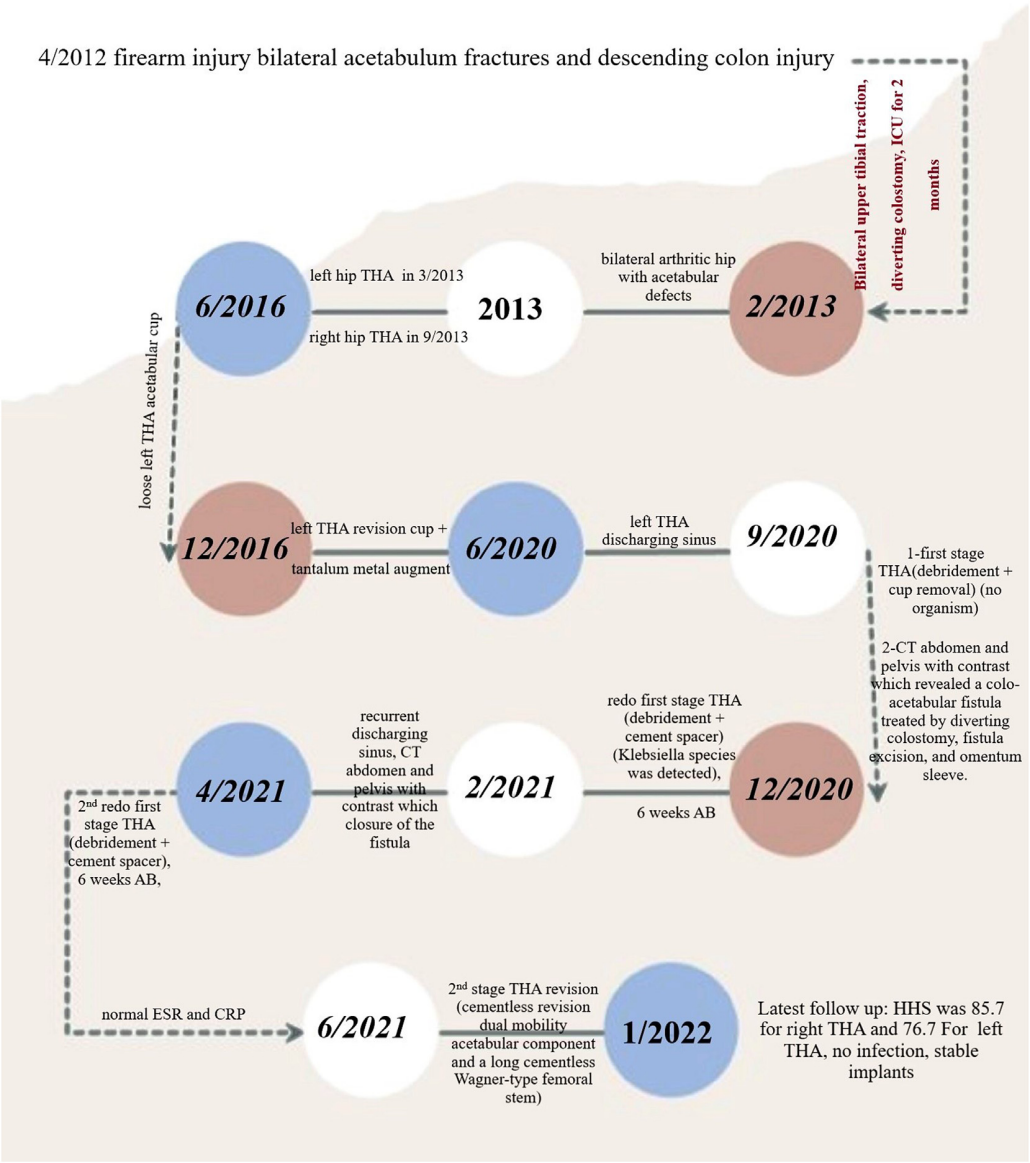
A timeline chart indicating the injuries and the surgeries the patient was subjected to.THA: total hip arthroplasty; ESR: erythrocyte sedimentation rate; HHS: Harris hip score; ICU: intensive care unit; CRP: C-reactive protein.

The treating general surgeon decided to perform a diverting colostomy, excised the fistula, and applied an omentum sleeve. Three months later (December 2020), the colostomy was closed; another debridement session to the left hip entailed stem removal, application of a cement spacer (Fig. 4A), and obtaining cultures which later showed contamination of the wound by *Klebsiella* species and antibiotics were given for 6 weeks. The patient presented with a discharging sinus from the wound in February 2021, consultation with a general surgeon for a fear of reformation of the fistulous tract, and after obtaining another CT scan with contrast which confirmed closure of the fistula in April 2021, another session of debridement and a cement spacer was performed (Fig. 4B). Antibiotics according to culture and sensitivity, were administered for another 6 weeks, during which the wound was fine, and the erythrocyte sedimentation rate and C-reactive protein showed declined to reach near-normal levels. A final THA revision was performed in June 2021 using a cementless revision dual mobility acetabular component and a long cementless Wagner-type femoral stem (Fig. 5A&B). At the latest follow-up in January 2022 (about 7 years for the right THA and 8 months for the left revision THA), the patient showed accepted hip function bilaterally (HHS was 85.7 (good) for the right hip and 76.7 (fair) or the left hip, which is accepted on both sides), no signs of infection; the implants were in a good position (indicated by restored and maintained hip center of rotation) (Fig. 5C). The history and surgical interventions are summarized in a timeline chart (Fig. 6).

## Discussion

Hip firearm injuries and their consequences, especially if combined with penetrating abdominal or pelvic injuries, pose a dilemma for trauma surgeons^3^^,^^4^^,^^6^; the choice had to be made between conservative and surgical management; the condition would be more complex if the injury was overlooked or neglected^16^.

After a firearm injury, the hip could be destroyed by one of the following possible causes: missed intraarticular fractures with joint incongruity leading to PTOA, concomitant joint infection after contamination in case of intestinal injuries, articular cartilage destruction, foreign body reaction, lead arthropathy, or synovitis.^3^^,^^17^

In a study by Pazarci et al.^2^ including 10 hips diagnosed with PTOA after gunshot injuries, and all of them were unilateral cases treated by cementless THA after a mean (4.9 ± 1.4) months post-injury, no reconstruction of the acetabulum was needed for any case, the HHS improved from a preoperative 25.2 points to 65.8 at the final follow-up, the worst functional outcomes were reported in patients with concomitant intestinal injury where all patients in this group had postoperative infection necessitating further surgical intervention. Like the previous study, Becker et al.^4^ reported postoperative infection in 4 of 5 patients who presented with concomitant transabdominal and hip firearm injury.

Özden et al.^3^ retrospectively evaluated 26 patients with a mean age of 31.5 years where THAs were performed to manage post-firearm hip arthritis after a mean of 15.7 months from the initial injury; all were unilateral cases, the HHS improved from a preoperative score of 53 to postoperative 80 points, infection occurred at an incidence of 23% (6 out of 26 hips).

Unilateral cases were reported in previous THA series performed for post-firearm hip injuries.^2^, ^3^, ^4^ Our case report was the first one to report bilateral PTOA caused by a single bullet to the best of our knowledge. Furthermore, we are aware of a coloarticular fistula after hip firearm injury case reported by Schiergens et al.^14^, a 25 years old male patient had a previous right THA after hip destruction caused by firearm injury; the authors diagnosed the patient with septic THA loosening, for which they performed a cement spacer; however, what pulled the attention of the authors to the colo-acetabular fistula was the abnormal organisms obtained from the surgical field, the diagnosed was later confirmed by CT pelvis with contrast. The authors treated the patient with an ileostomy and fistula closure using endoscopic over-the-scope clipping, followed by definitive THA after 3 months.

In the literature, a bowel-articular fistula (colo-articular or entero-articular) was documented in about 15 hips after various hip surgeries, including hip arthrodesis, primary THA, revision THA, hip resurfacing, Girdlestone resection arthroplasty, bipolar hemiarthroplasty, and after a cement spacer application.^7^, ^8^, ^9^, ^10^, ^11^, ^12^, ^13^, ^14^^,^^18^, ^19^, ^20^, ^21^, ^22^, ^23^, ^24^

In most of these cases, a fistula formation was attributed either to a pre-existing inflammatory bowel disease^18^^,^^19^, long-term steroid use^20^, or previous pelvic irradiation^21^^,^^22^; however, in revision THA surgeries, it was mainly attributed to the violation of the medial acetabular wall^8^^,^^12^. In a case reported by Dardas et al.^9^, where the entero-articular fistula occurred after the application of an antibiotic cement spacer, the authors attributed the thermal injury from the cement polymerization process as a possible contributing factor besides the medial bone defect. This led to the conclusion that forming a bowel-articular fistula after hip surgeries is mostly multifactorial, including factors related to the patient, the initial hip injury, and the status of the hip anatomy.

Furthermore, the time taken for the fistula to form and to be diagnosed varied dramatically among these reports and ranged from 2 days up to 30 years^7^, ^8^, ^9^^,^^11^, ^12^, ^13^, ^14^^,^^18^, ^19^, ^20^, ^21^, ^22^, ^23^, ^24^, while in the current case, a colo-articular fistula presented itself after 8 years from the initial injury. In most previous reports, a bowel-articular fistula was suspected if the patient had predisposing factors and persistent infection after excluding all other possible causes, mainly if atypical organisms were obtained from the surgical field.^14^

In the current case, we could not formulate a clear etiology or the starting time for the colo-articular fistula formation; however, some possible reasons could explain its occurrence. First, the fistula could be formed after the initial firearm injury attributed to the thermal injury of the bullet with further concomitant descending colon injury and acetabular fracture, with a possible subsequent friable adhesions formation between the bowel and the medial aspect of the acetabulum. Second, these adhesions could have been disrupted during the first revision surgery during the removal of the cemented acetabular cup or after reaming the acetabulum to implant a new acetabular cup, especially after a subsequent pelvis CT scan showed a violation of the medial wall of the acetabulum. Third, a cemented cup was used during the first and second revisions, and a thermal injury to the bowel during cement polymerization could have occurred.

Acetabular bone defect reconstruction using IBG allows for bone stock reconstruction and restoration of the hip center of rotation in complex primary and revision THA, with accepted long-term survival, even in younger patients^25^^,^^26^. Several techniques had been introduced for acetabular defect reconstruction during complex primary or revision THA, IBG since it was introduced by Slooff et al.^15^ had been used for biologically reconstructing acetabular bone defects, which showed promising results with long-term survivorship in many studies.^25^^,^^26^ Pazarci et al.^2^, and Özden et al.^3^, in their series, did not report the need for reconstructing the acetabulum; however, in the current case, we performed reconstruction of both the acetabulum on both sides using IBG and a cemented acetabular cup, which survived on the right side for about 8 years.

In conclusion, treating post-firearm hip arthritis by THA is challenging, especially in the case of neglected cases with the presence of an acetabular defect. Concomitant intestinal injury increases the risk of infection with the possibility of developing coloarticular fistula; the latter should be suspected if the patient presented with repeated infections, isolation of atypical organisms from the surgical field, and if the medial acetabular wall was violated. Working with a multidisciplinary team enables accurate diagnosis and management.

## Funding

This research did not receive any specific grant from funding agencies in the public, commercial, or not-for-profit sectors.

## Ethical statement

The ethical committee of our institution waived ethical approval for this case report as this was considered a part of the usual patients’ care.

## Declaration of competing interest

No conflict of interest for any author concerning this article.

## Author contributions

Ahmed M. Abdelaal carried out the case report conception and performed the arthroplasty-related surgeries. Ahmed Mohamed Ali is a colorectal surgeon who performed part of the surgeries.

Ahmed A. Khalifa, Mohammad Kamal Abdelnasser, Haisam Atta, and Mohamed MA Moustafa carried out data acquisition, assessment, literature search, and prepared the images.

Ahmed A. Khalifa and Mohammad Kamal Abdelnasser drafted the manuscript.

Ahmed Mohamed Ali and Ahmed M. Abdelaal and did the critical revision.

All authors read, discussed, and approved the final manuscript.
